# Consistent semantic representation learning for out-of-distribution molecular property prediction

**DOI:** 10.1093/bib/bbaf147

**Published:** 2025-04-10

**Authors:** Xinlong Wen, Hao Liu, Wenhan Long, Shuoying Wei, Rongbo Zhu

**Affiliations:** College of Informatics, Huazhong Agricultural University, No.1 Shizishan Street, Hongshan District, Wuhan, 430070, Hubei, People’s Republic of China; College of Informatics, Huazhong Agricultural University, No.1 Shizishan Street, Hongshan District, Wuhan, 430070, Hubei, People’s Republic of China; College of Informatics, Huazhong Agricultural University, No.1 Shizishan Street, Hongshan District, Wuhan, 430070, Hubei, People’s Republic of China; College of Informatics, Huazhong Agricultural University, No.1 Shizishan Street, Hongshan District, Wuhan, 430070, Hubei, People’s Republic of China; College of Informatics, Huazhong Agricultural University, No.1 Shizishan Street, Hongshan District, Wuhan, 430070, Hubei, People’s Republic of China

**Keywords:** consistent semantic, out-of-distribution, representation learning, molecular property prediction

## Abstract

Invariant molecular representation models provide potential solutions to guarantee accurate prediction of molecular properties under distribution shifts out-of-distribution (OOD) by identifying and leveraging invariant substructures inherent to the molecules. However, due to the complex entanglement of molecular functional groups and the frequent display of activity cliffs by molecular properties, the separation of molecules becomes inaccurate and tricky. This results in inconsistent semantics among the invariant substructures identified by existing models, which means molecules sharing identical invariant structures may exhibit drastically different properties. Focusing on the aforementioned challenges, in the semantic space, this paper explores the potential correlation between the consistent semantic–expressing the same information within different molecular representation forms–and the molecular property prediction problem. To enhance the performance of OOD molecular property prediction, this paper proposes a consistent semantic representation learning (CSRL) framework without separating molecules, which comprises two modules: a semantic uni-code (SUC) module and a consistent semantic extractor (CSE). To address inconsistent mapping of semantic in different molecular representation forms, SUC adjusts incorrect embeddings into the correct embeddings of two molecular representation forms. Then, CSE leverages non-semantic information as training labels to guide the discriminator’s learning, thereby suppressing the reliance of CSE on the non-semantic information in different molecular representation embeddings. Extensive experiments demonstrate that the consistent semantic can guarantee the performance of models. Overall, CSRL can improve the model’s average Receiver Operating Characteristic - Area Under the Curve (ROC-AUC) by 6.43%, when comparing with 11 state-of-the-art models on 12 datasets.

## Introduction

Accurate prediction of molecular properties plays a crucial role in computer-aided drug discovery. The key lies in precisely capturing comprehensive chemical semantics and extracting highly expressive molecular representations [1]. In recent years, molecular representation models [2, 3] have demonstrated impressive performance in experimental scenarios under the assumption that the training and test data adhere to the same distribution (independent and identically distribution, IID). However, in practical drug discovery scenarios, shifts in molecular data distribution often cause existing models to struggle with consistently capture invariant molecular representations under the complex entanglement patterns [4, 5], which significantly decline in the accuracy of molecular property prediction models.

**Figure 1:**
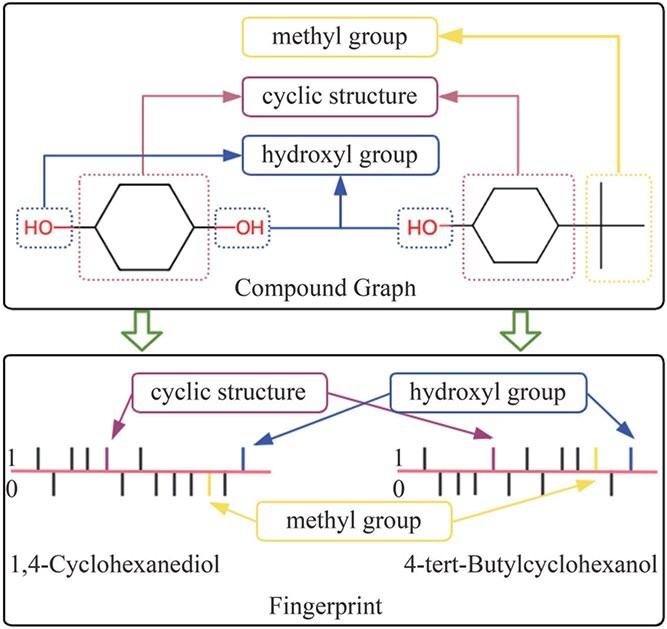
An example. Two molecules share substructures hydroxy (-OH) and 6C-ring, but they have different properties.

Given the fact that IID assumption lacks generalizable under distribution shifts, there has been a growing interest in extracting invariant molecular representation through various approaches, including the joint disentanglement of environment labels and prediction labels [6], environment interventions [7–11], latent space disentanglement [5], and information-theoretic based [12, 13]. However, these methods frequently overlook the activity cliffs and the indecomposability of complex molecules. There are the following possible challenges. First, molecular properties often exhibit activity cliffs, where structurally similar molecules may display significantly different properties [14, 15]. Second, molecular structures usually contains multiple interacting substructures, and analyzing these substructures in isolation makes it difficult to accurately predict molecular properties [16]. The complex interactions within molecules prevent models from simply separating the molecular structures into invariant and spurious substructures. Third, existing researches show that, without additional information, achieving generalization is theoretically impossible [6, 17]. Consequently, extracting invariant substructures from a single molecular representation form may result in inconsistent semantic, which inevitably hindering the extracted invariant substructures from fully express the high-level abstract features of the molecule. This impairs the generalization performance of the models. An illustrative example is provided in Fig. 1, where two molecules, 1,4-cyclohexanediol and 4-tert-butylcyclohexanol, share the same hydroxy (-OH) and scaffold patterns: 6C-ring [18]. Despite containing identical substructures, these molecules exhibit distinctly different hydrophilic or hydrophobic properties. At this point, if only a single molecular graph is considered, and both the hydroxy and 6C-ring are treated as the invariant substructures, the model will be unable to accurately distinguish between the two molecules. Hence, it remains open and tricky how to construct an invariant molecular representation model by utilizing the consistent semantics of molecules and their associated information (Such as molecular fingerprints) without decomposing the molecules.

Semantic-based approaches hold potential in addressing the aforementioned issues by abstracting different representation forms of the same entity into a task-oriented, unified high-level abstract representation [19]. For example, in natural language processing (NLP) field [20, 21], different words (such as “car” and “automobile”) can convey the same semantic information in specific contexts. In the field of fine-grained image recognition, models use textual prompts to guide the extraction of regions in images that express the same information as the prompts [22]. Similarly, although the hydroxy group in a molecule graph and the specific identifier in a molecular fingerprint are represented differently, they both convey the same semantic information of “hydrophilicity.” This observation inspired us to explore the potential of molecular consistent semantics in addressing the aforementioned issues. Specifically, unlike traditional molecular representation methods [23], semantic information represents the same message or symbol conveyed by different entities in specific scenarios and tasks [24]. In bioinformatics, molecular fingerprints are commonly adopted to describe the characteristics of molecules [25–27], which are typically represented as binary strings. In these strings, each bit (e.g. 0 or 1) represents a specific property of the molecule. By mapping 1,4-cyclonhexanediol and 4-tert-butylcyclohexanol to molecular fingerprints, we can also identify the portions of the fingerprint that convey the same information as molecular graph, such as the cyclic structure, hydroxyl, and methyl (as shown in the Fig. 1). Although the molecular graph and the molecular fingerprint differ in their representation forms (one being a visual chemical structure graph and the other is a digital code), they both convey the same semantic, information regarding “hydrophilicity” or “hydrophobicity.” Such semantic information conveyed through different molecular representation forms, is referred to as “Consistent Semantic” in this paper. Hence, a promising paradigm would be to extract molecular consistent semantics about specific attributes. However, in applying molecular consistent semantics to solve the out-of-distribution (OOD) generalization problem, we face the following questions:

Question 1: Is it feasible to use molecular consistent semantics to solve the OOD generalization problem in molecular property prediction?

Question 2: How can the semantic information between the encoded molecular graphs and molecular fingerprints be unified?

Question 3: How to accurately extract consistent semantics about specific attributes?

In response to these questions, this paper focuses on invariant molecular representation and proposes a consistent semantic extraction method without separating molecules, aiming to enhance the performance of OOD molecular property prediction models. The main contributions of this paper are summarized as follows:

(1) We explore the feasibility of using molecular consistent semantics to address the OOD generalization problem and further propose a consistency semantic representation learning framework (CSRL). CSRL comprises two modules: a contrastive learning-based semantic uni-code (SUC) module and an adversarial training-based consistent semantic extractor (CSE), thereby improving the model performance. To the best of our knowledge, this is the first framework that utilizes molecular consistent semantics, which extracted from different molecular representation forms, to solve the OOD generalization problem.

(2) To project molecular graphs and molecular fingerprints into the same semantic space, our SUC module adjusts embeddings by aligning those that lead to incorrect predictions with the correct one. SUC module balances the relationship between semantic information and other information across different molecular representation forms, thereby improving the model’s ability to express semantic information within various molecular representation forms.

(3) Our CSE incorporates a consistent semantic loss from the perspective of entropy by utilizing non-semantic information within different representation forms, to accurately extract consistent semantics between molecular graphs and their corresponding molecular fingerprints. Subsequently, the discriminator is trained using the consistent semantic loss to suppress the extracted non-semantic information, allowing CSE to more precisely extract consistency semantics from different molecular representation forms.

(4) We conduct extensive experiments on two real-world benchmarks, DrugOOD and ADMEOOD, using a total of 12 public datasets, along with an additional molecular property datasets, BBBP. Detailed results demonstrate that our model consistently and significantly outperforms various state-of-the-art (SOTA) baselines. Particularly, our method achieves up to 6.43% higher average ROC-AUC than those of 11 SOTA models.

## Related work

### Invariant graph representation learning

The purpose of invariant graph representation learning is to extract invariant substructures from data across different environments, thereby improving the model’s generalization capability in the presence of data shift [5, 8–13, 28]. LECI [6] adapted both environment and property labels available in the dataset to extract invariant substructures by jointly optimizing causal relationships. To mitigate the confusion effect in graph neural networks (GNNs), CAL [9] and CAL-Plus [10] introduced a causal attention learning strategy based on constructed causal graph, encouraging GNNs to focus more on causal structures. Similarly, DIR [11] and GSAT [8] utilized invariant substructures to tackle the OOD generalization problem. By intervening in the invariant substructures, multiple intervention graphs are generated to minimize invariant risk. To objectively validate the causal effects of causal substructures on predictions, DSE [28] introduced alternative variables for causal substructures through distribution interventions, enabling unbiased estimation of the relationships between causal substructures and labels. To jointly generate mixed environments and capture invariant patterns from mixed graphs, IGM [7] performed environment mixing and invariant substructures mixing simultaneously. This multi-level mixing approach generates a sufficiently diverse set of environments to encourage the model to capture the invariant patterns underlying the graphs. To address suboptimal problem of invariant features in complex molecules, iMoLD [5] employed a “first-encoding-then-separating” strategy. It maps original molecules into latent space before separation, thereby enhancing the model’s ability to represent invariant features. CIGA [13], developed from the perspective of graph generalization, constructed a structural causal model (SCM) for the graph classification and separates graph into causal and spurious parts. These methods aim to predict the labels of OOD data by identifying invariant substructures in molecules. However, due to the intricate entanglement of molecular structures and activity cliffs, existing methods for molecular invariant representation often struggle to accurately extract invariant substructures.

### Semantic representation

Semantic plays a pivotal role in various fields, such as semantic communication, image processing, molecular self-supervised learning, and so on. Numerous studies highlight that the original features contain much redundant information irrelevant to semantic discrimination. This redundancy can be processed by eliminating non-semantic information in similar samples [29–32]. To quantify semantic information, the vector quantized autoencoder (VQ-AE) [33] constructed a codebook-based semantic vector quantization autoencoder. VQ-AE not only processes and transmits information, but also enables the receiver to intuitively understand and analyze the semantic meaning of communication content. To effectively discard redundant features in the original features, U-DeepSC [34] employed a lightweight feature selection module to assess the importance of features. Then it adjusts the number of transmitted features based on varying channel conditions. To ensure the similarity between original and reconstructed images, CL [35] reduced the semantic distance between original and reconstructed images through contrastive learning, while maintaining the semantic distance between unrelated images. However, the aforementioned methods are not tailored for non-Euclidean data, such as molecules. In the field of molecular self-supervised learning, multi-granularity graph semantic ensemble via knowledge distillatioin (MGSE) [19] utilized distillation learning to guide multiple student models to learn different molecular substructures. MGSE used the knowledge learned from a pre-trained teacher model as high-level abstract features. However, this approach relies on clustering molecules, and when there is a shift in the data distribution, the model often fails to effectively cluster unseen data [36, 37], leading to OOD generalization problems. Notably, MGSE also decomposes molecules into several substructures.

Our work is sharply different from previous studies by (i) considering the feasibility of OOD molecular property prediction based on consistent semantics in semantic space and (ii) taking into account two molecular representation forms (i.e. graphs and fingerprints) to extract consistent semantic.

## Problem formulation

### Problem definition

We focus on the OOD generalization performance of molecular property prediction models. A molecular graph can be represented as $\mathcal{G} = (V, E)$, where $V$ and $E$ represent the atoms and the covalent bonds, respectively. Given a molecular graph training data $G^{train} = {(\mathcal{G}_{i}^{e}, Y_{i}^{e})}_{\mathcal{E}_{train}}$, where $\mathcal{G}_{i}^{e}$ is sampled from a known environment $\mathcal{E}_{train}$ and satisfies $\mathcal{E}_{train} \subseteq \mathcal{E}_{all}$. The key to the OOD generalization problem in molecular property prediction is to find a predictor $f: G \rightarrow Y$ such that the $f$ trained on $G^{train}$ can maintain stable predictive performance on $G^{all}$. Therefore, the goal of a generalized predictor $f$ trained on $G^{train}$ can be defined as follows:


(1) \begin{align*}& \min_{f} \max_{e \in \mathcal{E}_{all}} \mathbb{E}_{(\mathcal{G}_{i}, Y_{i}) \sim p(\mathcal{G}, Y \mid \mathbf{e} = e)} \left[l(f(\mathcal{G}), Y) \mid e \right],\end{align*}

where $l(\cdot , \cdot )$ represents the empirical loss function. However, since the training data only covers a very limited set of environments, most molecular property prediction models can only satisfy the following objective:


(2) \begin{align*}& \min_{f} \max_{e \in \mathcal{E}_{train}} \mathbb{E}_{(\mathcal{G}_{i}, Y_{i}) \sim p(\mathcal{G}, Y \mid \mathbf{e} = e)} \left[l(f(\mathcal{G}), Y) \mid e \right].\end{align*}

Comparing these two training objectives (Eq. 1 and Eq. 2), the methods for addressing the OOD generalization problem can be divided into two categories: (i) environment augmention [7–11], where the environments covered by the training set are as comprehensive as possible, i.e. $\mathcal{E}_{train} \rightarrow \mathcal{E}_{all}$; (ii) finding an invariant substructure $\mathcal{G}_{c}$ that is not affected by the environments [12, 13], i.e. $p(Y | \mathcal{G}_{c}, e) = p (Y | \mathcal{G}_{c})$. Due to the diversity of environments, it is challenging for models to automatically generate environments that perfectly encompass the training and validation data. Moreover, the intricate relationships between atoms and functional groups in molecules makes it difficult to completely divide a molecule into mutually independent invariant and spurious substructures. These make to solve Eq. 1 extremely difficult.

In this paper, we explore how to address the aforementioned issues from the perspective of molecular consistent semantics in semantic space. Consider the scenario depicted in Fig. 1: a molecule can be represented as a graph $\mathcal{G}$ and a fingerprint $\mathcal{F}$. Despite their representation forms are different, they convey consistent semantic $\mathcal{S}$ relevant to the molecular property prediction tasks. Therefore, our goal is to seek a molecular consistent semantic extractor $\Phi $ that satisfies:


(3) \begin{align*}& \mathcal{S} = \Phi(\mathcal{G}) = \Phi(\mathcal{F}).\end{align*}

### Semantic information on molecular property prediction

Semantic information represents the semantic characteristics conveyed by messages or symbols in specific tasks. Semantic entropy, originates from the analysis of NLP, aims to assess the depth of meaning within the data and capture its richness in the transmission process. Let $\bar{m}(\mathcal{S})$ represent the probability that $\mathcal{S}$ is accurately predicted by the predictor $m(\cdot )$ under all possible environments $e$, where $\mathcal{S} = \Phi (\mathcal{G})$. Then, semantic entropy $H_{\mathcal{S}}(\mathcal{G})$ can be defined as:


(4) \begin{align*}& H_{\mathcal{S}}(\mathcal{G}) = -log \bar{m}(\mathcal{S}).\end{align*}

Therefore, the higher the probability $\bar{m}(\mathcal{S})$, the lower the semantic entropy. The objective of the molecular property prediction model $\mathbb{S}$ can be defined as:


(5) \begin{align*}& \min_{m, \Phi} l(m(\mathcal{S}), Y), s.t. \mathcal{S} = \Phi(\mathcal{G}) = \Phi(\mathcal{F}), \min H_{\mathcal{S}}(\mathcal{G}).\end{align*}

### Feasibility analysis of consistent semantics

Definition 1. Different representation forms of a molecule contain the same semantic information, i.e. consistent semantics.

According to Definition.1, from the perspective of mutual information, $\mathcal{G}$ and $\mathcal{F}$ both convey consistent semantic $\mathcal{S}$. Therefore, we have: $I(\mathcal{S}; \mathcal{G}) = H(\mathcal{S}) - H(\mathcal{S} \mid \mathcal{G})$. Moreover, since $\mathcal{S} = \Phi (\mathcal{G})$, then $H(\mathcal{S} \mid \mathcal{G}) = 0$, i.e. $I(\mathcal{S}; \mathcal{G}) = H(\mathcal{S})$. Similarly, for $I(\mathcal{S}; \mathcal{F})$, we have:


(6) \begin{align*}& I(\mathcal{S}; \mathcal{F}) = I(\mathcal{S}; \mathcal{G}) = H(\mathcal{S}).\end{align*}

Furthermore, considering another scenario where both $\mathcal{G}$ and $\mathcal{F}$ serve as molecular representation forms and as inputs to the predictor $m(\cdot )$. We then have $I(\mathcal{S}; \mathcal{G}, \mathcal{F}) = H(\mathcal{S}) - H(\mathcal{S} \mid \mathcal{G}, \mathcal{F})$. Since $\mathcal{S} = \Phi (\mathcal{G}) = \Phi (\mathcal{F})$, $\mathcal{S}$ is determined when $\mathcal{G}$ and $\mathcal{F}$ are known, and thus $H(\mathcal{S} \mid \mathcal{G}, \mathcal{F}) = 0$. Therefore, we have:


(7) \begin{align*}& I(\mathcal{S}; \mathcal{F}) = I(\mathcal{S}; \mathcal{G}) = I(\mathcal{S} \mid \mathcal{G}, \mathcal{F}) = H(\mathcal{S}).\end{align*}

For molecular graph $\mathcal{G}$ and $\mathcal{F}$, we assume there exists other information $\mathcal{O}_{\mathcal{G}}$ and $\mathcal{O}_{\mathcal{G}}$ such that the following equations hold:


(8) \begin{align*} & G:= f_{gen}^{\mathcal{S}}(\mathcal{S}, \mathcal{O}_{\mathcal{G}}), \end{align*}

(9) \begin{align*} & F:= f_{gen}^{\mathcal{S}}(\mathcal{S}, \mathcal{O}_{\mathcal{F}}), \end{align*}

where $f_{gen}^{\mathcal{S}}(\cdot , \cdot )$ represents the mapping process that generates different molecular representation forms $\mathcal{G}$ and $\mathcal{F}$ by combining $\mathcal{S}$ with $\mathcal{O}_{\mathcal{G}}$ and $\mathcal{O}_{\mathcal{F}}$, respectively. From Equation. 7, we know that the reduction in uncertainty of $\mathcal{S}$ after knowing $\mathcal{G}$ is the same as the reduction in uncertainty of $\mathcal{S}$ after knowing both $\mathcal{G}$ and $\mathcal{F}$. This indicates that knowing one of $\mathcal{G}$ or $\mathcal{F}$, another does not provide any additional information about $\mathcal{S}$, i.e. we have:


(10) \begin{align*} & \mathcal{S} \perp \mathcal{O}_{\mathcal{G}}, \end{align*}

(11) \begin{align*} & \mathcal{S} \perp \mathcal{O}_{\mathcal{F}}. \end{align*}

Based on Equation. 5, 10 and 11, we have the following conclusions regarding consistent semantics:

- Consistent semantics ensure accurate predictions for OOD molecular property prediction models.
- Consistent semantics across different molecular representation forms is not influenced by other information, even if other information shifts.

These two conclusions about consistent semantic satisfy the sufficiency and invariance definitions of invariant features in the invariance theory [4]. Therefore, using consistent semantic features to alleviate OOD generalization problems is feasible, thereby addressing Question 1.

## Methods

**Figure 2:**
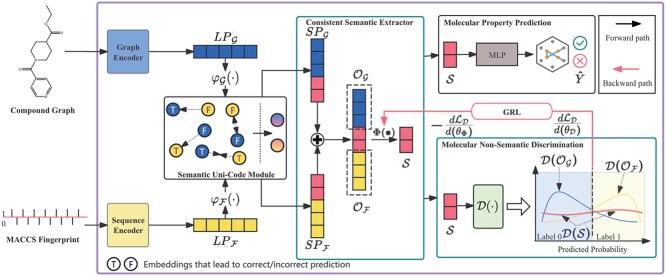
An overview of CSRL. Firstly, given a compound graph and its fingerprint, a SUC module is involved to obtain $SP_{\mathcal{G}}$ and $SP_{\mathcal{F}}$. Then a molecular non-semantic discriminator $D(\cdot )$ is designed to determine whether the molecular semantic extracted by Consistent Semantic extractor $\Phi (\cdot )$ contains any extraneous information. Finally, these semantics are utilized to predict the property $\hat{Y}$ of molecules.

**Figure 3:**
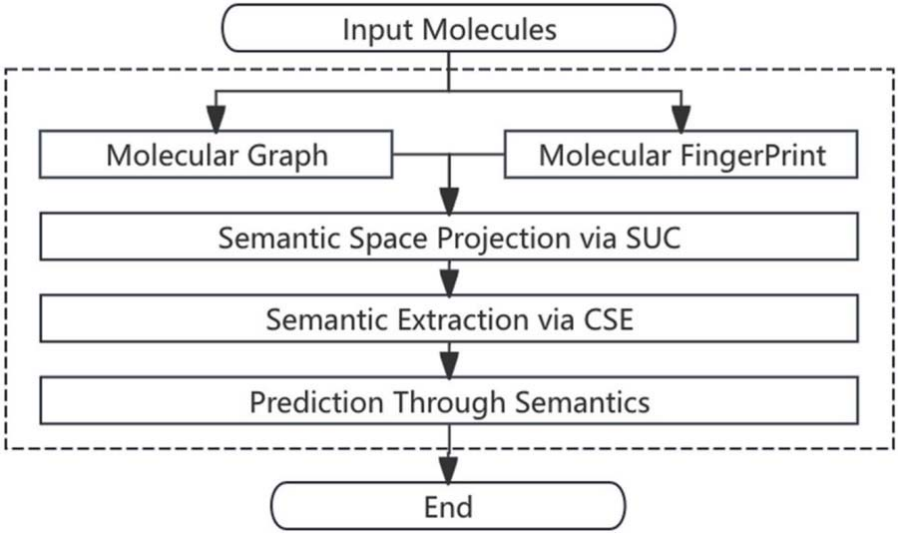
The outlines of CSRL.

This section presents the details of our proposed CSRL. Fig. 2 provide an overview of CSRL, which mainly consists of three steps: (i) using an encoder and a SUC module obtain the unified representation of molecular graphs and fingerprints in the semantic space (Providing a solution to Question 2); (ii) extracting molecular consistent semantics through an adversarial training based CSE (Providing a solution to Question 3); (iii) utilizing the extracted consistent semantics for supervised learning to optimize the overall process. The flow chart of our proposed method is shown in Fig. 3.

### Project feature space into semantic space with semantic uni-code module

Current mainstream methods [5, 10, 13] explicit or implicit separate molecules into invariant and spurious substructures. In contrast, CSRL does not require the separation of molecules but instead approaches the problem from a semantic perspective, thereby avoiding the challenge of non-independence that arises during the separation process.

Given a molecular graph $\mathcal{G}$ and its corresponding MACCS fingerprint $\mathcal{F}$, we first adapt an graph encoder $f_{h}(\cdot )$ to encode $G$. Simultaneously, we encode $\mathcal{F}$ using a projection function $f_{p}(\cdot )$. This dual encoding scheme generates latent representations $LP_{\mathcal{G}}$ and $LP_{\mathcal{F}}$ in the feature space, corresponding to $\mathcal{G}$ and $\mathcal{F}$, respectively.


(12) \begin{align*} & LP_{\mathcal{G}} = f_{h}(\mathcal{G}) \in \mathbb{R}^{|B| \times d}, \end{align*}

(13) \begin{align*} & LP_{\mathcal{F}} = f_{p}(\mathcal{F}) \in \mathbb{R}^{|B| \times d}, \end{align*}

where $B$ denotes the batch size, and $d$ corresponds to the dimensionality of the representation in the feature space.

Since $LP_{\mathcal{G}}$ and $LP_{\mathcal{F}}$ originate from different molecular representation forms, there is an inconsistency issue in the semantic space between $LP_{\mathcal{G}}$ and $LP_{\mathcal{F}}$. To mitigate this discrepancy, we constructed a SUC module, which aims to further balance the semantic information $\mathcal{S}$ and other information $\mathcal{O}_{\mathcal{G}}$ and $\mathcal{O}_{\mathcal{F}}$ within $LP_{\mathcal{G}}$ and $LP_{\mathcal{F}}$. Consequently, the SUC module facilitates enhanced expression of consistent semantic in the semantic space.

We first use cross-entropy loss to constrain the representations of $\mathcal{G}$ and $\mathcal{F}$ in feature spaces, $LP_{\mathcal{G}}$ and $LP_{\mathcal{F}}$. The constrain loss $\mathcal{L}^{\mathcal{G}}$ and $\mathcal{L}^{\mathcal{F}}$ can be formally defined as:


(14) \begin{align*} & Y_{\mathcal{G}} = \varphi_{\mathcal{G}}(LP_{\mathcal{G}}),\ \ \ \qquad\qquad\qquad\qquad\qquad\qquad\qquad\qquad\qquad \end{align*}

(15) \begin{align*} & Y_{\mathcal{F}} = \varphi_{\mathcal{F}}(LP_{\mathcal{F}}),\ \ \ \qquad\qquad\qquad\qquad\qquad\qquad\qquad\qquad\qquad \end{align*}

(16) \begin{align*} & \mathcal{L}^{\mathcal{G}} = -\frac{1}{N} \sum_{i=1}^{N} [y_{i} \log(Y_{\mathcal{G}}) + (1 - y_{i})\log(1 - Y_{\mathcal{G}})], \quad y_{i} \in Y, \end{align*}

(17) \begin{align*} & \mathcal{L}^{\mathcal{F}} = -\frac{1}{N} \sum_{i=1}^{N} [y_{i} \log(Y_{\mathcal{F}}) + (1 - y_{i})\log(1 - Y_{\mathcal{F}})], \quad y_{i} \in Y, \end{align*}

where $\varphi _{\mathcal{G}}{(\cdot )}$ and $\varphi _{\mathcal{F}}{(\cdot )}$ denote Multilayer Perceptron (MLP) classifier. $Y$ represents the ground-truth molecular labels corresponding to the molecules.

Next, $LP_{\mathcal{G}}$ and $LP_{\mathcal{F}}$ in the feature space undergo projection into the semantic space to enable semantic correction, thereby unifying the semantic information within $LP_{\mathcal{G}}$ and $LP_{\mathcal{F}}$. To operationalize this process, we formulate a contrastive loss for optimizing CSE. For the selection of positive and negative pairs, we have the following discussion:

When $Y_{\mathcal{G}} = Y_{\mathcal{F}} \neq Y$, we consider that $LP_{\mathcal{G}}$ and $LP_{\mathcal{F}}$ do not contain any valid information, so the distance between $LP_{\mathcal{G}}$ and $LP_{\mathcal{F}}$ is maximized. When $Y_{\mathcal{G}} = Y$ and $Y_{\mathcal{F}} \neq Y$, or $Y_{\mathcal{G}} \neq Y$ and $Y_{\mathcal{F}} = Y$, we consider that the embedding that leads to correct prediction contains semantic, and the incorrectly one needs to be corrected by referring to the correctly predicted embedding. When $Y_{\mathcal{G}} = Y_{\mathcal{F}} = Y$, both embeddings contain semantic information.

Finally, contrastive loss is used to achieve the above process and we can obtain two types of representations ($SP_{\mathcal{G}}$ and $SP_{\mathcal{F}}$) in the semantic space, formally expressed as:


(18) \begin{align*} & \mathcal{L}_{cl} = --\log \frac{\sum_{(LP_{\mathcal{G}}, LP_{\mathcal{F}}) \sim \mathbb{P}} \exp\left(\frac{\cos(LP_{\mathcal{G}}, LP_{\mathcal{F}})}{\tau}\right)}{\sum_{(LP_{\mathcal{G}}, LP_{\mathcal{F}}) \sim \mathbb{B}} \exp\left(\frac{\cos(LP_{\mathcal{G}}, LP_{\mathcal{F}})}{\tau}\right)}, \end{align*}

(19) \begin{align*} & SP_{\mathcal{G}} = SUC(LP_{\mathcal{G}}) \in \mathbb{R}^{|B| \times d},\qquad\qquad\qquad\qquad\qquad \end{align*}

(20) \begin{align*} & SP_{F} = \text{SUC}(LP_{\mathcal{F}}) \in \mathbb{R}^{|B| \times d},\ \ \ \quad\qquad\qquad\qquad\qquad \end{align*}

where $\mathbb{P}$ indicates the embedding pair set that causes an incorrect prediction in either $Y_{\mathcal{G}}$ or $Y_{\mathcal{F}}$, $\mathbb{B}$ denotes the embedding pair set of all molecules. The temperature coefficient $\tau $ is used to adjust the smoothness of the distribution. Consequently, the molecular representations $SP_{\mathcal{G}}$ and $SP_{\mathcal{F}}$ can be obtained, which balance the semantic $S$ and other information $\mathcal{O}_{\mathcal{G}}$ and $\mathcal{O}_{\mathcal{F}}$.

### Consistent semantic extractor in semantic space

After obtaining two types of representation ($SP_{\mathcal{G}}$ and $SP_{\mathcal{F}}$) in the semantic space, consistent semantic can be extracted from them. Importantly, our model does not involve separating the molecular into invariant and spurious parts. Instead, we consider the entire molecular features to extract consistent semantics. This approach offers several advantages: (i) The complete molecular features can maximize the model’s understanding of the molecular property prediction task. (ii) It avoids feature misalignment caused by splitting already unified features.

We propose an adversarial training framework to extract consistent semantics. First, we construct a consistent semantic extractor $ \Phi (\cdot ) $ to extract the common semantic features from $ SP_{\mathcal{G}} $ and $ SP_{\mathcal{F}} $. Given the molecular representations $ \text{SP}_{G} $ and $ \text{SP}_{F} $, the fused representation $ CP$ can be obtained through the $Concat(\cdot , \cdot )$ operation:


(21) \begin{align*}& CP = \sigma(Concat(SP_{\mathcal{G}}, SP_{\mathcal{F}})) \in \mathbb{R}^{|B| \times d},\end{align*}

where $ \sigma (\cdot ) $ represents the projection function, which aligns the dimensions of $CP $ with the dimension of $ SP_{\mathcal{G}} $ and $SP_{\mathcal{F}} $. Subsequently, the consistent semantic representation $ \mathcal{S} $ of the molecule is obtained via $ \Phi (\cdot ) $, formally expressed as:


(22) \begin{align*}& S = \Phi(CP) \in \mathbb{R}^{|B| \times d}.\end{align*}

Then, to determine whether the extracted consistent semantic $\mathcal{S}$ contains other information ($ \mathcal{O}_{\mathcal{F}} $ and $ \mathcal{O}_{\mathcal{G}} $) or not, we designed a molecular non-semantic discriminator $ \mathcal{D}(\cdot ) $. However, due to the inaccessibility of $ \mathcal{O}_{\mathcal{F}} $ and $ \mathcal{O}_{\mathcal{G}} $, training a $ \mathcal{D}(\cdot ) $ is particularly challenging. This necessitates the development of an indirect optimization strategy for discriminator training.

It is evident that $SP_{\mathcal{G}} $ and $ SP_{\mathcal{F}} $ inherently encapsulate the complete $ \mathcal{O}_{\mathcal{F}} $ and $ \mathcal{O}_{\mathcal{G}} $. Therefore, the other information labels of $SP_{\mathcal{G}} $ and $SP_{\mathcal{F}} $ are set to 1 and 0, respectively. This constructed dataset facilitates the training of $ \mathcal{D}(\cdot ) $ through cross-entropy minimization. Then, we have:


(23) \begin{align*} & \mathcal{L}_{\mathcal{G}} = -\frac{1}{N} \sum_{i=1}^{N} \log(\mathcal{D}(\mathcal{G}_{i})),\qquad \end{align*}

(24) \begin{align*} & \mathcal{L}_{\mathcal{F}} = -\frac{1}{N} \sum_{i=1}^{N} \log(1 - \mathcal{D}(\mathcal{F}_{i})), \end{align*}

where $ N $ is the number of samples, and $ \mathcal{G}_{i} $ and $ \mathcal{F}_{i} $ represent the $ i $-th samples in the constructed dataset.

However, ground-truth labels for $ \mathcal{S} $ cannot be obtained accurately in advance, and $ \mathcal{D}(\cdot ) $ cannot calculate the prediction loss for $ \mathcal{S} $. Therefore, we construct a consistent semantic loss from the perspective of entropy. The key insight is that while $ \mathcal{D}(\cdot ) $ should reliably classify $ SP_{\mathcal{G}} $ and $ SP_{\mathcal{F}} $, it should exhibit maximum uncertainty when predicting the label of the extracted $ \mathcal{S} $. Specifically, we hope that $ \mathcal{D}(\cdot ) $ predicts the probabilities of $ \mathcal{S} $ belonging to the 0 or 1 to both be 0.5, at which point the prediction probability entropy is 1. Suppose the probability that $ \mathcal{D}(\cdot ) $ predicts $ \mathcal{S} $ belongs to label 0 is $ p $, then the probability that it belongs to label 1 is $ (1 - p) $. The prediction probability entropy $H(S)$ of $ \mathcal{D}(\cdot ) $ is then computed as:


(25) \begin{align*}& H(S) = -p \log_{2} (p) - (1 - p) \log_{2} (1 - p).\end{align*}

Finally, the consistent semantic loss $\mathcal{L}_{\mathcal{S}}$, which optimizes the $ \mathcal{D}(\cdot ) $ toward maximum prediction uncertainty for $\mathcal{S}$, can be formally described as follows:


(26) \begin{align*}& \mathcal{L}_{\mathcal{S}} = \frac{1}{N} \sum_{i=1}^{N} (1 - H(\mathcal{S})),\end{align*}

where $\lambda $ represents the strength of gradient reversal, preventing $\Phi{\cdot }$ from overfitting to $ SP_{\mathcal{G}} $ or $ SP_{\mathcal{F}} $. Here, we set $\lambda $ to 1.

### Learning objective of CSRL

The optimization objective of the CSRL model consists of three parts: the (i) SUC loss of the SUC module, (ii) the consistent semantic extraction loss of the CSE module, and (iii) the task prediction loss for molecular property prediction task.

(1) SUC loss of the SUC module. The SUC module aims to project molecular representation from the feature space into the semantic space, while balancing the relationship between semantic information and other information through correction and unified coding operations. The loss function $\mathcal{L}_{SUC}$ can be described as the sum of the constraint loss and contrastive loss:


(27) \begin{align*}& \mathcal{L}_{SUC} = \mathcal{L}^{\mathcal{G}} + \mathcal{L}^{\mathcal{F}} + \mathcal{L}_{cl}.\end{align*}

(2) Consistent semantic extraction loss of the CSE. The CSE employs adversarial training of $ \mathcal{D}(\cdot ) $ and $ \Phi (\cdot ) $ to extract more accurate molecular consistent semantic $ \mathcal{S} $. Specifically, to facilitate adversarial learning process, Gradient Reversal Layer (GRL) [38] is used to guide $\Phi (\cdot )$ in learning consistent semantics that are irrelevant to $ \mathcal{O}_{\mathcal{F}} $ and $ \mathcal{O}_{\mathcal{G}} $.

During the forward pass, the GRL does not alter the input data, allowing CSE outputs the consistent semantics $ \mathcal{S} = \Phi (CP) $, which are passed to the discriminator $ \mathcal{D}(\cdot ) $. However, during backpropagation, the GRL multiplies the gradient of the discriminator’s loss by a negative constant $ \lambda $, effectively reversing the gradient propagated to the feature extractor.

This process can be formalized as:


(28) \begin{align*}& \frac{d\mathcal{L}_{\mathcal{D}}}{d(\mathbf{\theta_\Phi})} = -\lambda \frac{d\mathcal{L}_{\mathcal{D}}}{d(\theta_{\mathcal{D}})},\end{align*}

where $ \lambda $ is the gradient reversal coefficient, and in this paper, we set $\lambda $ to 1. The overall loss of the CSE can be described as the cumulative sum of the losses:


(29) \begin{align*}& \mathcal{L}_{CSE} = \mathcal{L}_{\mathcal{G}} + \mathcal{L}_{\mathcal{F}} + \mathcal{L}_{\mathcal{S}}.\end{align*}

(3) Task prediction loss: The goal is to accurately extract task-related consistent semantic that robustly generalize to OOD molecular property prediction problems. In Equation 5, $ \min _{m, \Phi } l(m(\mathcal{S}), Y) $ can be described as the cross-entropy loss as:


(30) \begin{align*}& \mathcal{L}_{pred} = -\frac{1}{N} \sum_{i=1}^{N} [y_{i} \log(m(\Phi(CP))) + (1 - y_{i})\log(1 - m(\Phi(CP)))].\end{align*}

Finally, the learning loss of CSRL can be defined as the weighted sum of the above losses:


(31) \begin{align*}& \mathcal{L}_{total} = \lambda_{1} \mathcal{L}_{pred} + \lambda_{2} \mathcal{L}_{SE} + \lambda_{3} \mathcal{L}_{SUC},\end{align*}

where $ \lambda _{1} $, $ \lambda _{2} $, and $ \lambda _{3} $ are the hyper-parameters to control the weights of $\mathcal{L}_{SUC}$, $\mathcal{L}_{SE}$, and $\mathcal{L}_{pred}$. The specific algorithm details of CSRL are shown in Algorithm 1.




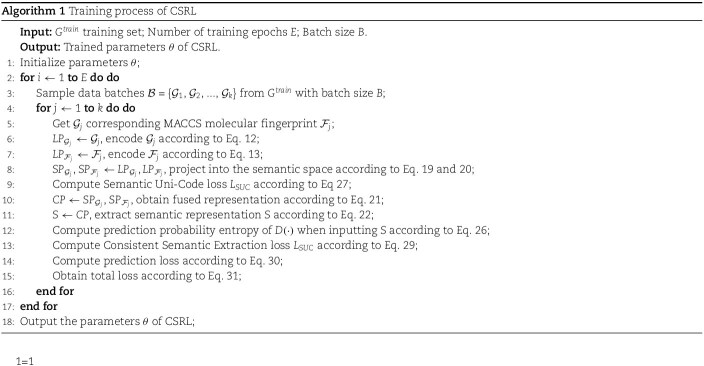



## Experiments

In this section, we first provide a brief introduction of datasets and baselines. Second, we introduce the experimental setting. Third, we compare our approach with 11 SOTA methods on ADMEOOD [39], DrugOOD [40] benchmarks and BBBP dataset [41]. Next, we analyze the contribution of each module in the CSRL model to the overall performance. After that, we investigate the impact of parameter selection for different modules on model performance. Following this, we use t-SNE [42] to visualize the proposed consistent semantics, thereby understanding the semantic space we proposed and the distribution of molecules within this space. Finally, we provide a case study to further interpret how the model makes predictions.

### Experimental setup

Datasets. Two real-world benchmarks, ADMEOOD and DrugOOD, are utilized to validate the performance of the proposed scheme. Additionally, to further verify the generalization ability of CSRL, we evaluated it on another type of benchmarks, MoleculeNet [41].

- DrugOOD is an OOD benchmark for AI-assisted drug discovery, focusing on affinity prediction between macromolecules and small molecules in drug-target binding tasks. This benchmark includes three distinct domains: assay, scaffold, and size, covering two measurement types: IC50 and EC50. Additionally, each combination of domain and measurement type is further divided into three different noise levels, resulting in a total of 18 datasets. In this paper, we selected IC50 and EC50 measurement types of three domain (assay, scaffold, and size), totalling 6 datasets. Additionally, to test the model’s performance under different degrees of distribution shift and evaluate its adaptability to such shifts, we selected the EC50 measurement type with three levels of distribution shift: core, refined, and general.
- ADMEOOD is a systematic benchmark designed for predicting OOD molecular properties, which is based on 27 properties across five dimensions of absorption, distribution, metabolism, excretion, and toxicity (ADMET). By applying Concept Conflict Drift (CCD), the data are divided into IID and OOD subsets. ADMEOOD includes two distinct domains: core and scaffold, covering three measurement types: EC50, KI, and POTENCY, totalling six datasets. In this paper, we selected EC50 and KI measurement types of two domains (core and scaffold), totalling four datasets.
- MoleculeNet is a widely used benchmark for molecular machine learning, designed to provide standardized datasets and evaluation frameworks for the fields of cheminformatics and drug discovery. It encompasses various types of tasks, including molecular property prediction, toxicity assessment, protein–ligand binding affinity prediction, and more, integrating multiple public datasets such as Tox21, BBBP, ESOL, and FreeSolv. In this paper, BBBP and Tox21 are utilized, and for Tox21, a single task is selected

For both benchmarks, the explanations of each domain are shown as follows:

- Assay (core). Assay is an experimental approach used to evaluate or determine specific molecular characteristics. Variations in assay conditions and target molecules often result in significant differences in the activity values measured across different assays. When samples are tested within the same assay, they are considered as part of a single environment. However, shifting to a different assay can cause a notable distribution shift due to the considerable differences in experimental methods and testing targets between assays.
- Scaffold. Molecules sharing the same molecular scaffold are grouped within the same domain, as the scaffold forms the core structure that imparts key bioactive properties to the molecule. The molecular characteristics can vary significantly between different scaffolds, leading to distinct environments. A distribution shift occurs when there is a change in the molecular scaffold, reflecting the differences in molecular properties associated with different scaffolds.
- Size. Molecular size is determined by the total number of atoms it contains, which is a fundamental structural characteristic of molecular graphs. A domain is defined by grouping samples with the same molecular size. Consequently, a distribution shift can occur when the size of the molecules changes, allowing us to evaluate the model’s performance on molecules that differ significantly in atomic composition.

Baselines. We thoroughly compare our CSRL model against ERM [43] and two groups of tailored for OOD learning baselines:

- Non-decompositional methods. IRM [44], V-REx [45], MolOOD [4], which use environment labels to mitigate the interference caused by environmental changes on the model.
- Decompositional methods. CIGA [13], CAL-Plus [10], DIR [11], iMolD [5], IGM [7] which separate molecular graphs or features into invariant parts that are unaffected by the environment and spurious parts that vary with environmental changes. Additionally, to investigate the impact of the backbone on the model, we modified the backbone of the IGM model by replacing GIN with GCN and GAT.

Metric. We note that the datasets provided by the DrugOOD and ADMEOOD benchmarks exhibit extreme class imbalance between positive and negative samples. As discussed in Appendix.1 of the supplementary materials, such imbalance can lead to misleading results when using accuracy (ACC) as an evaluation metric, as it fails to adequately reflect model performance on minority classes [46, 47]. Therefore, for OOD molecular property prediction tasks, ROC-AUC is a more appropriate and robust metric. ROC-AUC is widely adopted in existing works for its ability to evaluate model performance independently of class distribution, making it particularly suitable for imbalanced datasets [5, 7, 13].

Considering the characteristics of molecular property prediction tasks and the significant imbalance between positive and negative samples in the dataset, ROC-AUC is adapted to evaluate model performance.

Evaluation and important details. We use the default dataset split proposed in each benchmark. For BBBP and tox21 datasets, we randomly split the data into training, validation, and test sets with a 6:2:2 ratio. Since the Tox21 dataset contains multiple tasks and the labels for each task have missing values, we selected one specific task and removed the data points with missing labels. Otherwise, for all models, during the preprocessing process, we only removed molecules that could not be converted into graph structures. The statistics of the datasets are in Table 1. All models are evaluated on the datasets from DrugOOD and ADMEOOD benchmarks using the official benchmark code. All baseline models use the recommended parameters from the official code. For the purpose of comparison, we chose the following fixed settings for all models: embedding dimensions: 128, batch size: 128, learning rate: 1e-3, epochs: 30, Backbone architecture: GIN (graph isomorphism network). We performed three independent runs with different random seeds. In each independent run, we recorded the model that performed best on the validation set and subsequently evaluated its performance on the test set. Based on the results of these three training processes, we report the mean and standard deviation values of ROC-AUC.

**Table 1:** Dataset statistics

**Benchmark**	**Dataset**		**Task**	**Metric**	**#Train**	**#Val**	**#Test**
DrugOOD	IC50	Assay	Binary classification	ROC-AUC	34 953	19 475	19 463
		Scaffold	Binary classification	ROC-AUC	22 025	19 478	19 480
		Size	Binary classification	ROC-AUC	37 497	17 987	16 761
	EC50	Assay	Binary classification	ROC-AUC	4978	2761	2725
		Scaffold	Binary classification	ROC-AUC	2743	2723	2762
		Size	Binary classification	ROC-AUC	5189	2495	2505
ADMEOOD	EC50	Core	Binary classification	ROC-AUC	12 129	298	295
		Scaffold	Binary classification	ROC-AUC	11 723	583	583
	KI	Core	Binary classification	ROC-AUC	23 487	434	431
		Scaffold	Binary classification	ROC-AUC	31 110	583	583
MoleculeNet	BBBP		Binary classification	ROC-AUC	1223	408	408
	Tox21		Binary classification	ROC-AUC	4359	1453	1452

### Performance comparison

The experimental results of the comparative models on the DrugOOD benchmark, as well as the performance results on the ADMEOOD benchmark, BBBP and Tox21 datasets, are summarized in Table 2 and Table 3, respectively. It is noteworthy that DrugOOD benchmark uses covariant shift to construct IID and OOD datasets, which is relatively simple, and thus all methods perform well on DrugOOD benchmark. In contrast, ADMEOOD benchmark is constructed using samples with contradictory labels, resulting in a significant shift between the IID and OOD datasets [39], and generally weaker model performance on ADMEOOD datasets. Our method CSRL achieves the best performance on 8 of the 12 datasets and ranks second on the other two datasets, with an average ROC-AUC boost of 6.43% compared to the previous SOTA methods. While DIR shows significantly lower performance compared to other methods. Specifically, DIR relies on environmental interventions to extract invariant features. When dealing with molecules, which have complex entanglement patterns and activity cliffs, using other molecular spurious substructures as an intervention environment often introduces additional noise, leading to a decline in model performance. In contrast, our methods is able to achieve the best performance on most of the datasets without decomposing molecules, which indicates that the proposed consistent semantics in the semantic space is effective for OOD molecular property prediction. From the perspective of average performance, we observe that iMoLD, specifically designed for molecules, ranks second, which indicates that strategies for identifying invariant features in the feature space are effective.

**Table 2:** Evaluation performance on DrugOOD benchmarks. The best is marked with boldface and the second best is with underline

**Method**	**DrugOOD-IC50**			**DrugOOD-EC50**		
	**Assay**	**Scaffold**	**Size**	**Assay**	**Scaffold**	**Size**
ERM	70.55(0.55)	67.30(1.12)	**65.67(0.18)**	73.62(4.11)	64.81(1.11)	64.43(1.46)
IRM	67.83(1.26)	63.81(0.25)	63.03(0.41)	72.82(2.44)	63.51(1.05)	63.04(1.01)
VREX	68.48(0.73)	63.50(0.35)	63.46(0.49)	74.30(3.04)	64.55(1.92)	63.09(0.32)
DIR	68.05(0.51)	64.37(0.72)	63.24(0.48)	66.78(6.30)	57.90(5.12)	56.11(6.17)
MoleOOD	70.86(1.72)	66.38(0.72)	64.03(1.29)	70.39(1.73)	64.45(1.81)	61.38(2.11)
CIGA	68.59(0.60)	64.07(1.15)	63.22(0.63)	73.40(5.85)	63.24(1.80)	63.08(0.99)
iMoLD	70.11(0.32)	66.29(1.78)	65.31(0.04)	74.46(2.16)	65.95(0.77)	62.73(1.13)
CAL-plus	70.98(0.50)	66.88(0.81)	64.01(0.72)	68.04(1.31)	64.06(2.09)	56.06(3.15)
IGM-GIN	69.49(0.93)	65.92(0.58)	64.60(0.65)	72.61(6.63)	63.04(6.90)	63.24(1.72)
IGM-GCN	67.44(0.62)	64.64(0.91)	63.38(0.33)	65.58(0.86)	62.24(0.58)	60.82(1.99)
IGM-GAT	67.44(0.62)	62.20(0.76)	63.03(0.86)	64.71(1.44)	60.25(0.61)	58.11(1.53)
**CSRL**	**75.04(1.12)**	**69.47(0.28)**	65.55(0.12)	**75.58(1.29)**	**68.35(0.30)**	**64.48(0.73)**

**Table 3:** Evaluation performance on ADMEOOD benchmarks and BBBP, tox21 datasets. The best is marked with boldface and the second best is with underline

**Method**	**ADME-OOD-EC50**		**ADME-OOD-KI**		**BBBP**	**tox21**
	**Core**	**Scaffold**	**Core**	**Scaffold**		
ERM	40.50(0.58)	53.33(2.56)	49.98(1.13)	46.16(11.94)	88.69(0.50)	78.39(1.69)
IRM	37.14(1.60)	54.33(3.36)	45.84(2.28)	42.62(4.71)	79.76(13.4)	76.54(1.66)
VREX	41.31(2.47)	55.78(4.61)	45.23(1.90)	39.37(5.83)	78.79(9.94)	76.40(2.30)
DIR	39.32(0.59)	49.82(2.40)	48.37(2.87)	47.33(3.83)	74.14(27.9)	68.37(5.78)
MoleOOD	45.63(2.79)	54.69(3.25)	42.51(0.13)	46.11(5.17)	87.97(0.47)	79.64(2.98)
CIGA	37.83(3.21)	55.51(5.93)	48.11(0.60)	42.59(6.27)	85.14(12.3)	76.90(3.27)
iMoLD	40.14(2.95)	57.81(1.12)	49.45(2.17)	46.05(1.77)	87.92(1.34)	79.27(2.20)
CAL-plus	52.59(2.62)	45.44(6.34)	48.59(1.50)	**50.30(1.93)**	85.14(12.3)	74.38(2.06)
IGM-GIN	44.92(1.78)	51.76(7.49)	48.12(3.56)	36.56(1.85)	87.62(0.90)	75.32(2.04)
IGM-GCN	41.71(1.59)	62.23(3.47)	45.04(1.91)	38.86(1.64)	86.89(0.46)	76.45(1.56)
IGM-GAT	38.57(5.75)	53.87(6.34)	47.56(1.58)	41.17(2.36)	80.95(1.42)	80.79(1.31)
**CSRL**	**53.03(0.64)**	**71.26(1.55)**	**51.65(4.22)**	47.91(1.67)	**90.37(0.94)**	**82.11(1.25)**

The results on the EC50-assay datasets under different noise levels (core, refined, and general), which represent the degree of distribution shift between the training set and the test set, are reported in Table 4. From the experimental results, it can be observed that CSRL outperforms ERM by up to 8.13% and surpasses the SOTA method iMoLD by 0.88% on average. This demonstrates that CSRL has stronger adaptability to distribution shifts. Notably, as the noise level increases, the performance of all models shows a certain degree of degradation.

**Table 4:** Evaluation performance on EC50-assay with different degrees of distribution shift.

**Method**	**DrugOOD-EC50-Assay**		
	**Core**	**Refined**	**General**
ERM	73.62(4.11)	70.25(0.48)	68.53(0.75)
IRM	72.82(2.44)	70.90(1.32)	68.55(0.20)
VREX	74.30(3.04)	70.37(1.94)	68.28(0.63)
DIR	66.78(6.30)	63.30(1.22)	63.15(1.05)
MoleOOD	70.39(1.73)	67.11(0.98)	67.96(0.12)
CIGA	73.40(5.85)	71.07(1.10)	68.05(0.51)
iMoLD	74.46(2.16)	71.08(0.76)	68.87(0.27)
CAL-plus	68.04(1.31)	71.27(1.15)	68.63(0.84)
IGM-GIN	72.61(6.63)	69.74(0.72)	68.68(0.57)
IGM-GCN	69.44(0.16)	69.18(1.80)	67.73(0.93)
IGM-GAT	67.44(0.62)	68.32(1.4)	61.78(3.26)
CSRL	**75.58(1.29)**	**71.32(0.07)**	**69.39(1.50)**

The best is marked with boldface and the second best is with underline.

Overall, projecting molecular representations from the feature space to the semantic space and utilizing the consistent semantics extracted from the two types of molecular representation forms is a reasonable and effective approach.

### Ablation study of components

Through the above experiments, we validated the effectiveness of CSRL. In this section, we discuss the contributions of the main components in CSRL. We design the following variants of CSRL:

- CSRL without semantic extractor (w/o cse).
- CSRL without SUC module (w/o suc).
- CSRL without semantic extractor and SUC module (none).

Table 5 presents the experimental results on the assay-split and scaffold-split of DrugOOD-EC50 datasets, core-split, and scaffold-split of ADMEOOD-EC50 datasets. It can be observed that utilizing the molecular fingerprints as additional features, even without using any invariant learning methods, CSRL (None) achieves an average ROC-AUC performance of up to 63.78%. This finding underscores the effectiveness of combining molecular graphs with molecular fingerprints in reducing the difference between IID and OOD data. When CSRL lacks SUC module (w/o suc), the ROC-AUC performance drops by 3.85%. This is because the different representations of the molecules are not uniformly encoded and projected into the semantic space, making it difficult for CSE to correctly extract the consistent semantics. Notably, on the DrugOOD-EC50-scaffold dataset, the performance of CSRL (w/o suc) is even 0.25% lower than that of the base model. This performance drop may be due to the presence of a large amount of non-semantic overlapping parts in this dataset, leading to the extraction of some irrelevant information within the consistent semantics, thereby affecting the model’s performance. When CSRL lacks consistent semantic extractor (w/o cse), the performance drops by 3.6%. In this case, although the model can effectively encodes different representation forms of the molecules into the semantic space, the presence of other information within the semantic prevents the model from identifying the consistent semantics, reducing the model’s expressive capability. In conclusion, the combined use of SUC and CSE can improve the accuracy of consistent semantic extraction, helping to enhance the model’s generalization.

**Table 5:** Ablation studies. The “None” and “Full” rows represent the results for our basic framework and full CSRL model

**Ablation**	**DrugOOD-EC50**		**ADMEOOD-EC50**		**Avg**
	**Assay**	**Scaffold**	**Core**	**Scaffold**	
w/o cse	74.83(1.15)	68.12(0.26)	48.55(2.05)	66.88(1.76)	64.60
w/o suc	74.57(1.29)	67.44(0.39)	46.74(2.00)	68.95(2.77)	64.43
None	74.36(1.54)	67.61(0.13)	44.44(3.79)	68.72(1.27)	63.78
Full	**75.58(1.29)**	**68.35(0.30)**	**53.03(0.64)**	**71.26(1.55)**	**67.01**

### Hyperparameter sensitivity analysis

**Figure 4:**
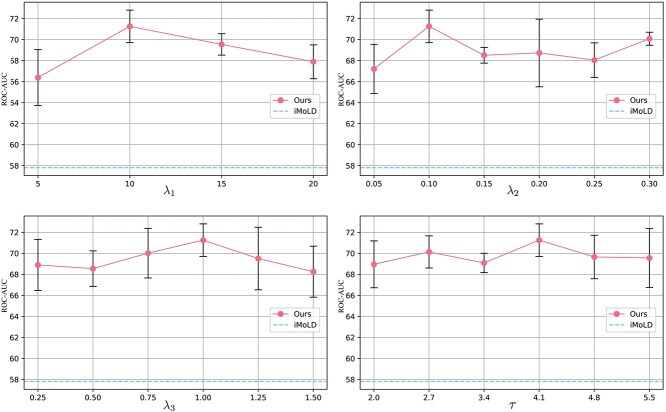
Hyper-parameter sensitivity analysis on the ADMEOOD-ec50-scaffold dataset. For each sensitivity study, we fix other hyperparameters with the values selected from the previous experiments.

Taking the scaffold-split dataset from ADMEOOD-EC50 as an example, we conduct extensive experiments to investigate the hyper-parameter sensitivity, and the results are presented in Fig. 4. Overall, CSRL demonstrated robustness under different hyper-parameter settings and consistently outperformed the SOTA invariant molecular representation method, iMoLD, in feature space. We observed that as $\lambda _{1}$, $\lambda _{2}$, and $\lambda _{3}$ increased, the model’s performance tends to improve first and then decreased slightly. This indicates that $\mathcal{L}_{pred}$, $\mathcal{L}_{CSE}$, and $L_{SUC}$ are effective and can improve performance within a reasonable range. The impact of the temperature coefficient $\tau $, which adjusts the degree of distribution smoothing, is not very significant and does not exhibit a clear trend. Additionally, we also observed that when $\lambda _{1}$ is too small or too large, there is a sharp decline in CSRL’s performance. When $\lambda _{1}$ is too small, it fails to ensure the completeness of the extracted consistent semantic. Additionally, a large $\lambda _{1}$ causes the model to overemphasize the completeness of semantics while neglecting the accurate discrimination of consistent semantic, resulting in a decline in performance.

### The distribution of molecules in semantic space

**Figure 5:**
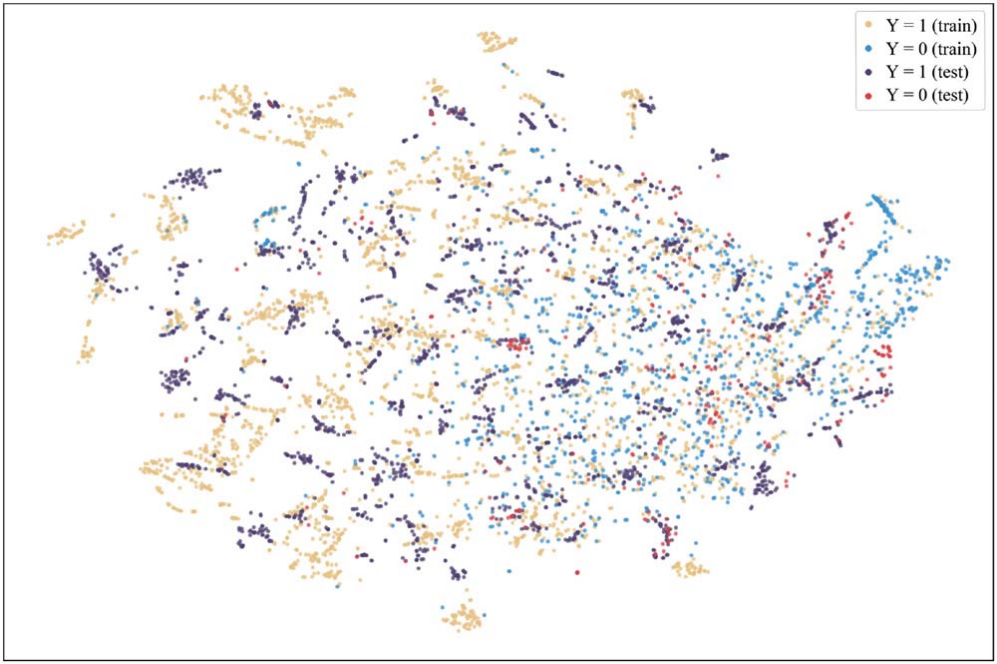
ERM, assay, and Wasserstein distance 0.89.

**Figure 6:**
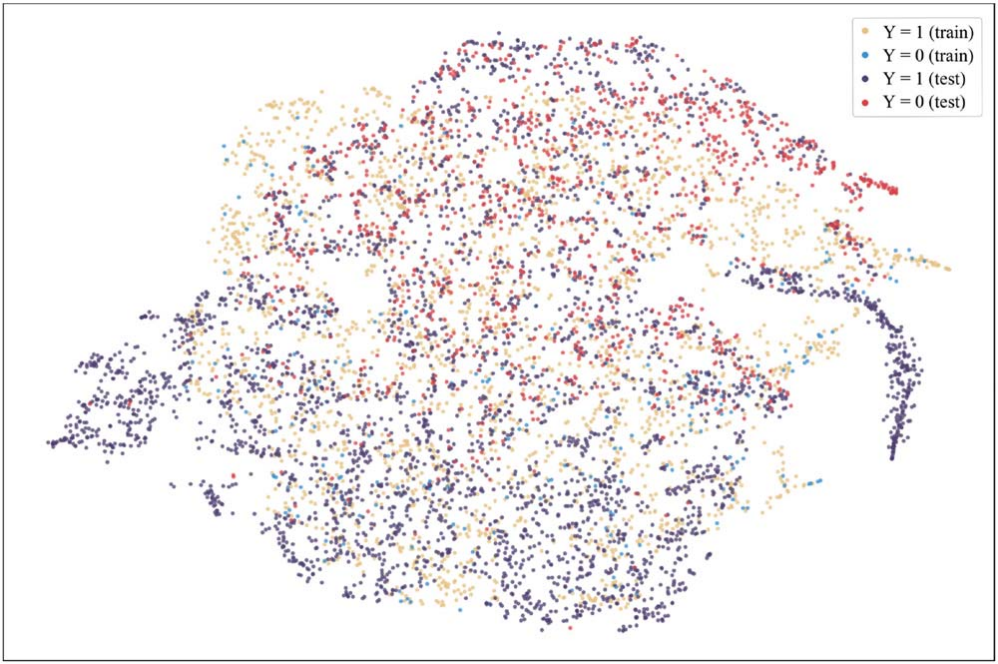
iMoLD, assay, and Wasserstein distance 1.12.

**Figure 7:**
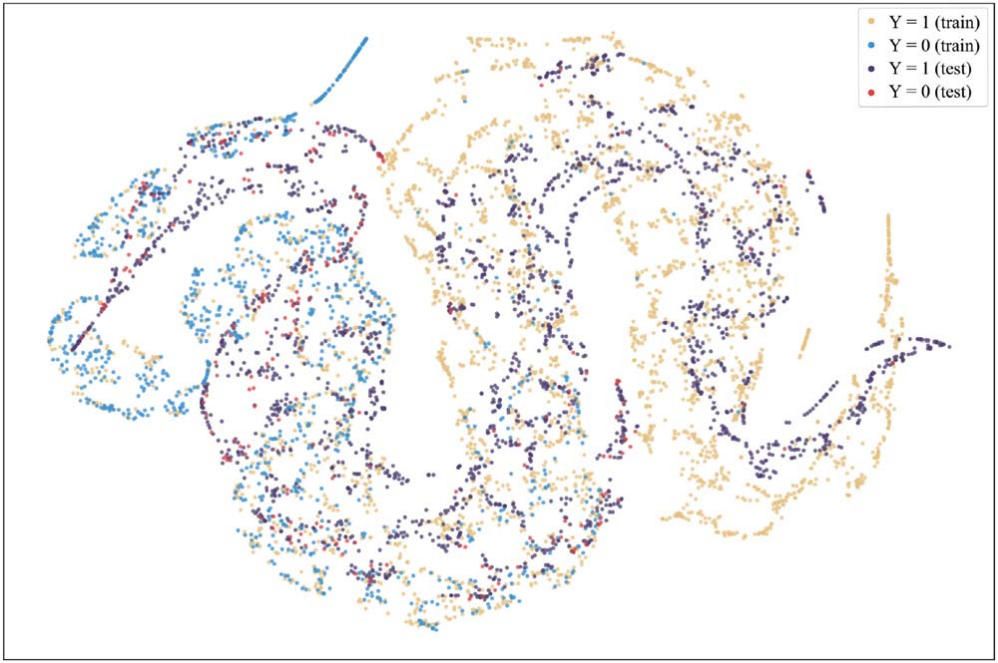
CSRL, assay, and Wasserstein distance 0.87.

**Figure 8:**
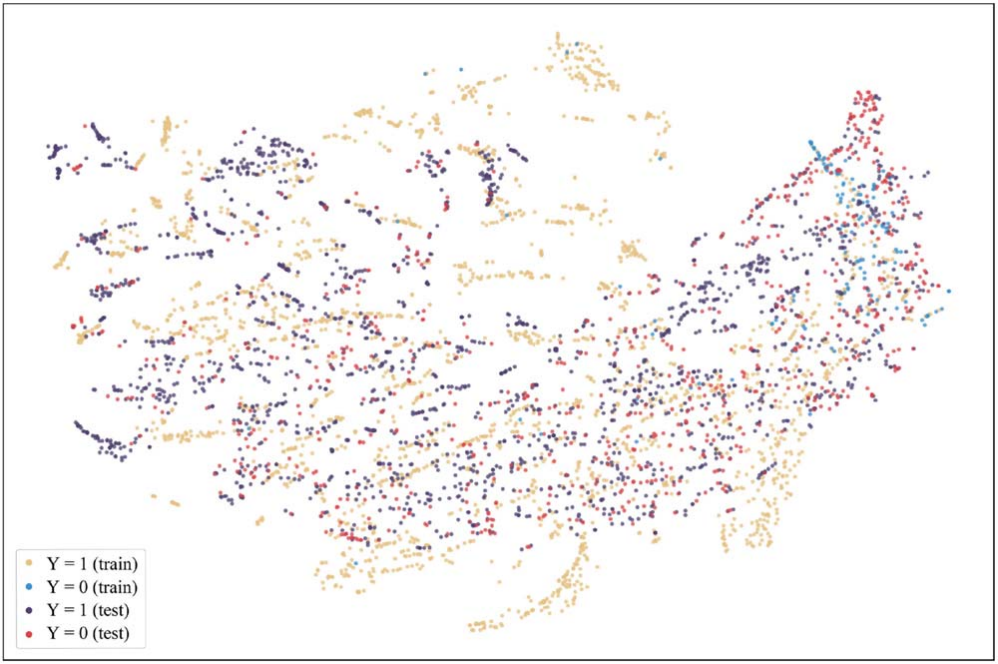
ERM, scaffold, and Wasserstein distance 2.75.

**Figure 9:**
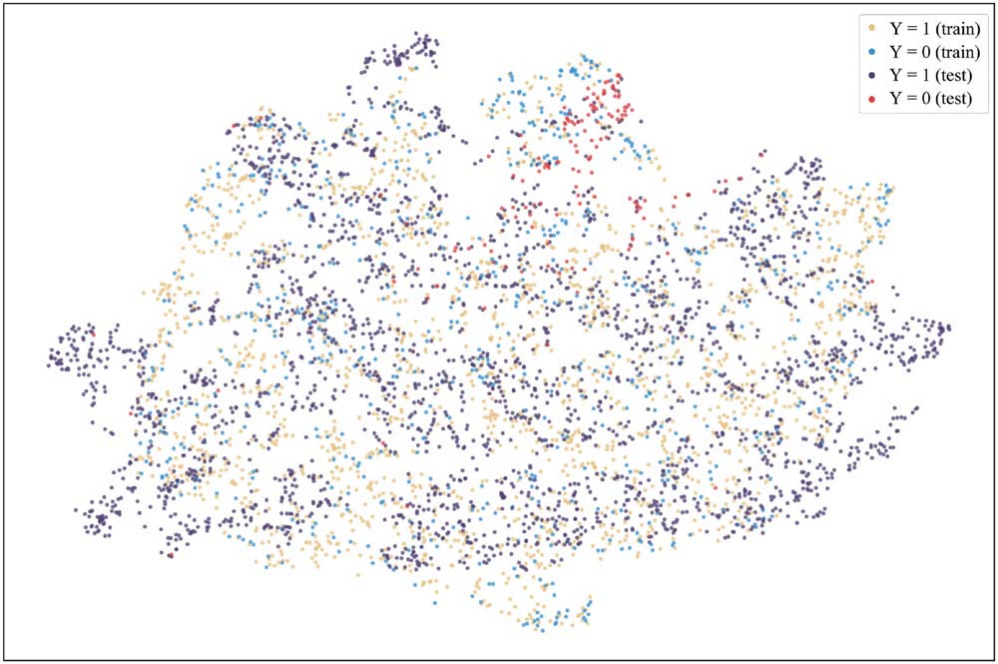
iMoLD, scaffold, and Wasserstein distance 0.95.

**Figure 10:**
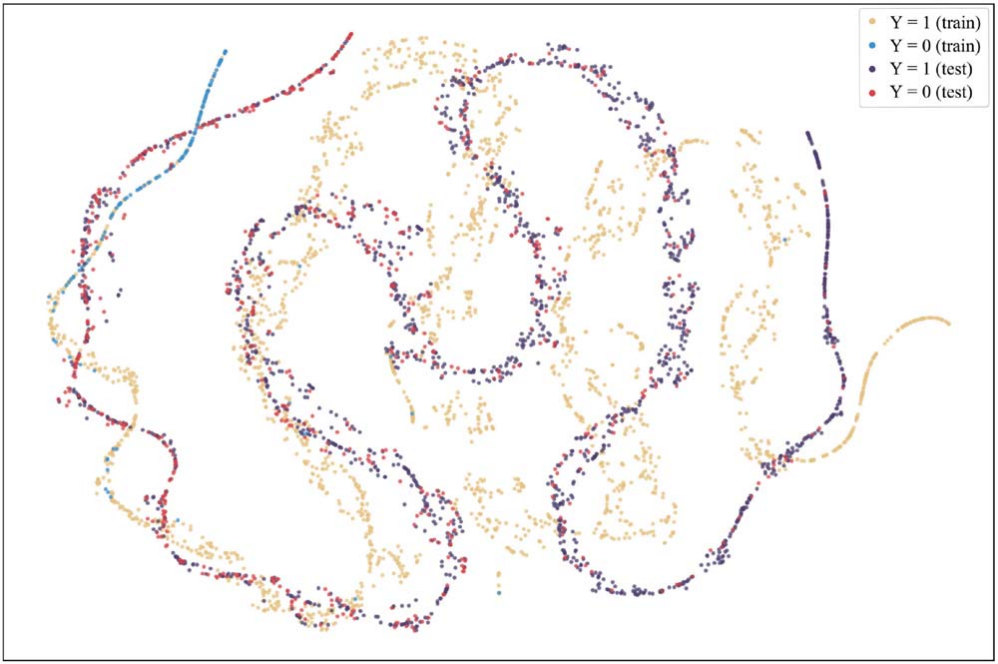
CSRL, scaffold, and Wasserstein distance 0.79.

**Figure 11:**
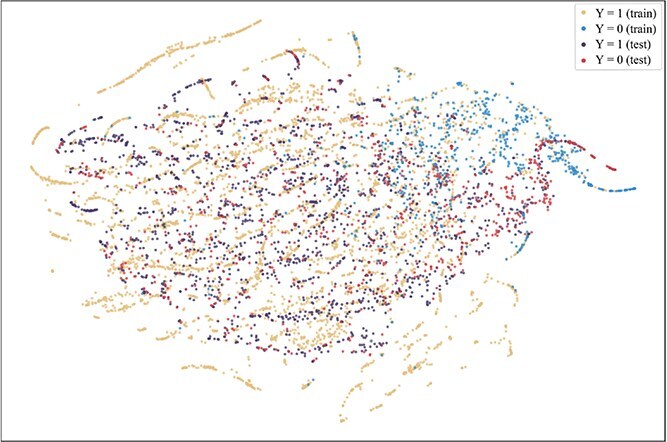
ERM, size, and Wasserstein distance 0.86.

**Figure 12:**
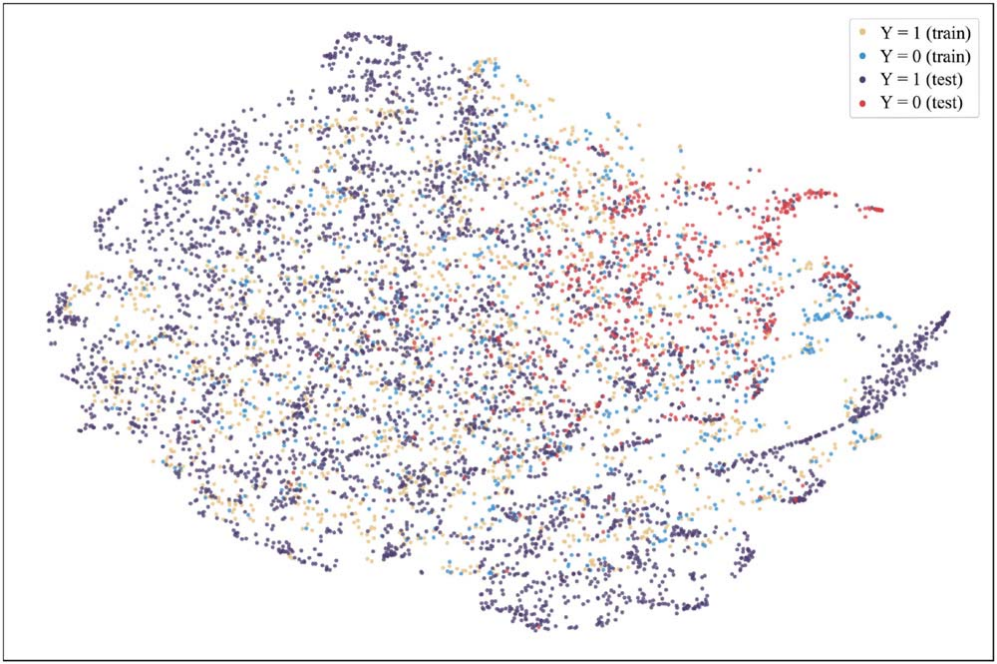
iMoLD, size, and Wasserstein distance 0.56.

**Figure 13:**
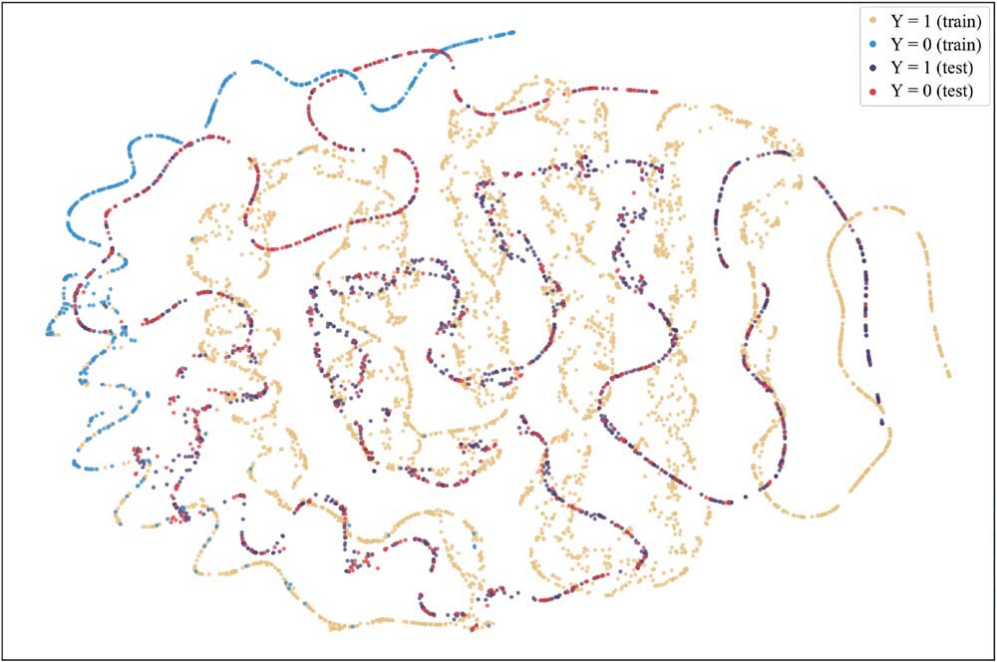
CSRL, size, and Wasserstein distance 0.45.

To further explore the reasons for the superior performance of our method and the advantages brought by introducing the consistent semantics, t-SNE [42] is used to visualize the projection of extracted consistent semantic $\mathcal{S}$ on training and test sets when the model achieved the best performance on the validation set on the Assay, Scaffold, and Size-split of DrugOOD-EC50 datasets, as shown in Figs. 5–13. We also visualize the results of some baselines, including the original ERM (Figs. 5, 8 and 11), iMoLD (Figs. 6, 9 and 12). We additionally compute the first-order Wasserstein distance [48] between the training and test sets, to quantify the dissimilarity in the consistent semantic distribution across varying environments. In Figs. 5–13, it can be observed that compared to ERM and iMoLD, CSRL reduced the Wasserstein distance by 53.11% and 19.77% on average, respectively. It indicates that CSRL is effective in identifying semantic information across different environments. On the other hand, from the visualization results of our model (Figs. 7, 10 and 13), it can be intuitively observed that the distribution of the training and test sets shows significant similarity, which may be due to the positive effect brought by the introduction of the innovative strategy of consistent semantics.

**Figure 14:**
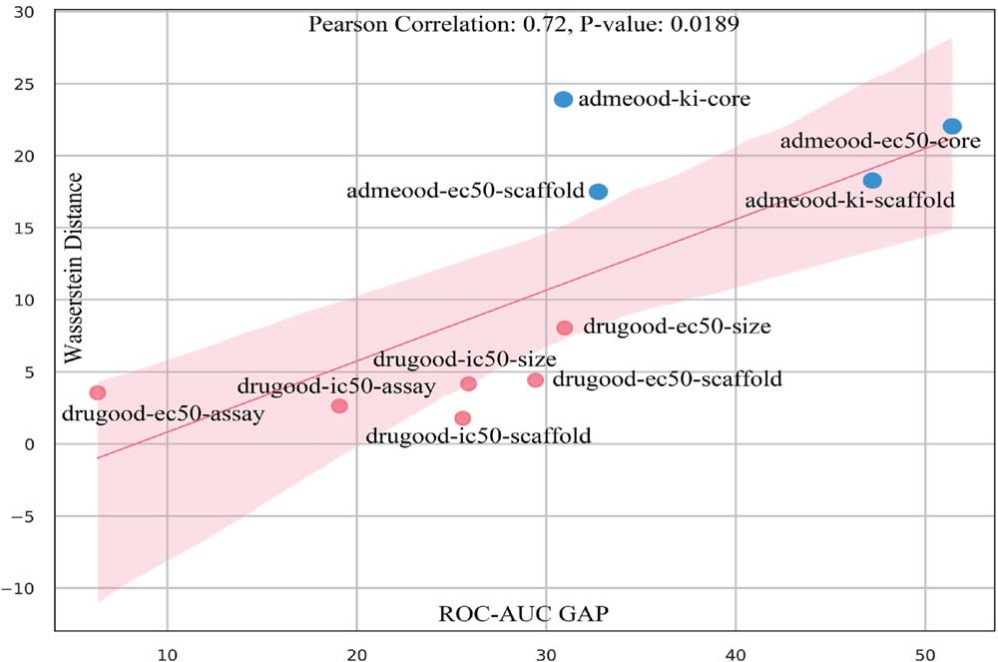
Visualization between Wasserstein distance and ROC-AUC gap.

To further investigate the relationship between ROC-AUC performance and Wasserstein distance, we conducted additional experiments. Specifically, we trained an ERM model to map both the training and test sets into a feature space. We then calculated the ROC-AUC performance gap between the training and test sets, as well as the Wasserstein distance between their feature distributions, as shown in Fig. 14. Finally, we computed the Pearson correlation coefficient between the ROC-AUC gap and the Wasserstein distance, which yielded a value of 0.72 with a P-value of 0.0189. The Pearson correlation coefficient measures the linear relationship between two variables, ranging from -1 to 1. A value of 0.72 indicates a strong positive correlation, suggesting that as the Wasserstein distance increases, the ROC-AUC gap also tends to increase. The P-value of 0.0189, which is below the conventional significance threshold of 0.05, confirms that this correlation is statistically significant. This result demonstrates that the distribution shifts can be evaluate by Wasserstein distance. Notably, the ADMEOOD benchmark exhibits significantly larger Wasserstein Distance compared to the DrugOOD benchmark, which is consistent with the description of ADMEOOD as a more challenging OOD benchmark [39].

### Case study

**Figure 15:**
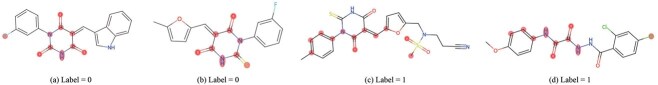
Visualization of atoms with the top 40% of attention weights in assay and scaffold split of the DrugOOD-EC50 dataset.

To illustrate the interpretability power of CSRL, we selected two molecules each from the datasets on assay and scaffold split of DrugOOD-EC50, input them into the same model, and present the top 40% of atoms with the highest attention weights. Fig. 15 displays the graphical structures of these molecules along with their corresponding labels, where the molecules in Fig. 15(a) and (b) are from the assay-split, and those in Fig. 15(c) and (d) are from the scaffold-split. We observe that when the labels are the same, CSRL consistently focuses on similar molecular topological structures. For instance, in Fig. 15(a) and (b), although the molecules are from different samples in the Assay dataset, the model still captures structural patterns highly correlated with the labels. Notably, when the labels differ, as shown in Fig. 15(a) and (c), despite the two molecules sharing the same topological structure, CSRL does not focus on their common structure, even though this structure is significantly correlated with label 0. Instead, the model shifts its attention to other discriminative patterns. This demonstrates that CSRL can effectively extract semantic information from molecules, avoiding over-reliance on fixed topological patterns, thereby better capturing stable features relevant to the target labels.

## Conclusion

In this work, we propose a novel molecular CSRL framework, which achieves SOTA performance on the DrugOOD, ADMEOOD benchmarks, as well as on other molecular property datasets, BBBP and Tox21. Furthermore, we evaluate CSRL under different degrees of distribution shift, showing its superior performance compared to baseline methods. Through ablation experiments, we have demonstrated the effectiveness of the proposed SUC and CSE, which can address the issues of inconsistent projection between different molecular representation forms and the difficulty in extracting consistent semantics, respectively. Notably, the consistent semantics extracted by CSRL shows significant consistency in distribution by t-SNE. Therefore, our model can ensure excellent generalization performance under data distribution shifts. In addition, we explore the interpretability of CSRL, demonstrating its ability to capture meaningful molecular features that contribute to robust predictions. Considering the limitations of information contained in molecular fingerprints, our future work will explore diverse molecular representations to extract consistent semantics. Additionally, we will collaborate with other disciplines to validate our proposed model in wet-lab experiments, ensuring its feasibility and practical effectiveness. We hope that this framework will promote research on the use of consistent semantics against distribution shift.

**Key Points**

- We explore the feasibility of using molecular consistent semantics to address the out-of-distribution (OOD) generalization problem and further develop a consistency semantic representation learning (CSRL) framework.
- Semantic uni-code module is proposed to adjust embeddings by aligning those that lead to incorrect predictions with the correct one, to improve semantic representation across different molecular forms.
- Consistent semantic extractor utilizes consistent semantic loss to suppress non-semantic information, enabling precise extraction of consistent semantics between molecular graphs and fingerprints.
- Extensive experimental results show that CSRL is highly competitive compared to the state-of-the-art methods.

## Supplementary Material

CSRL-supplementary_bbaf147

## Data Availability

The full datasets and the source codes for CSRL are freely available at https://github.com/Chertuion/CSRL.
