# Supplementary material for: Consistent semantic representation learning for out-of-distribution molecular property prediction
Source: Brief Bioinform. 2025 Apr 10;26(2):bbaf147. doi: 10.1093/bib/bbaf147 (PMC11982020; doi:10.1093/bib/bbaf147)
Supplement: CSRL-supplementary_bbaf147 [file csrl-supplementary_bbaf147.pdf]

Briefings in Bioinformatics, 2022, pp. 1–1

doi: DOI HERE
Advance Access Publication Date: Day Month Year
Paper

Supplementarty Data

PAPER

Consistent Semantic Representation Learning for Out-of-Distribution Molecular Property Prediction

Xinlong Wen[1], Hao Liu[1], Wenhan Long[1], Shuoying Wei[1] and Rongbo Zhu[1,*]

[1]College of Informatics, Huazhong Agricultural University, People's Republic of China.
[*]Corresponding author. Rongbo Zhu, E-mail: rbzhu@mail.hzau.edu.cn.

Abstract

In this supplementary, we discuss why we use the ROC-AUC as the primary metric instead of ACC in out-of-distribution molecular property prediction.

Appendix.1

The performance on accuracy metric

Table S1 summarizes the experimental results of the comparative models on DrugOOD, ADMEOOD benchmarks and another molecular properties prediction task, BBBP. Overall, the model we proposed still outperforms all the compared methods with a narrow performance gap.

Additionally, we observed that many datasets exhibit multiple best-performing results with identical performance, such as in the ADMEOOD-EC50-core and KI-scaffold datasets. Particularly in the KI-scaffold dataset, the variance of the best performance is 0. To explain this phenomenon, we first analyzed the distribution of positive and negative samples in the test sets across all datasets. Furthermore, we calculated the accuracy (ACC) when the model predicts all samples as the majority class (i.e., the most frequent label in the imbalanced dataset), as shown in Table S2.

From Table S2, it can be observed that class imbalance is highly prevalent in out-of-distribution molecular property prediction tasks. Taking the admeood-ki-scaffold dataset as an example, the positive-to-negative sample ratio reaches 1:11.2. Such extreme class imbalance allows the model to achieve an accuracy of 91.78% simply by predicting all samples as the negative class (0), thereby masking the model's true performance. Notably, this phenomenon is entirely consistent with our results in DIR and MoleOOD from S1, further validating the pervasiveness of the class imbalance issue.

Additionally, from Table S1, it can be observed that on the drugood and admeood benchmarks, the performance gap between the best and second-best models is often very small. Such minimal performance differences make it difficult for accuracy (ACC) to effectively distinguish the superiority of models. Therefore, in out-of-distribution molecular property prediction tasks, using ACC as an evaluation metric has significant limitations.

Table S1. Evaluation performance on DrugOOD, ADMEOOD benchmarks and another molecular properties prediction task, BBBP. The best is marked with **boldface** and the second best is with underline.

Method	DrugOOD-IC50			DrugOOD-EC50			ADME-OOD-EC50		ADME-OOD-KI		BBBP	Avg
	Assay	Scaffold	Size	Assay	Scaffold	Size	Core	Scaffold	Core	Scaffold		
ERM	83.61(0.08)	77.19(0.11)	**73.89(0.05)**	90.45(1.14)	73.35(0.22)	(70.21(1.05))	**76.38(1.52)**	84.11(1.59)	66.13(1.84)	87.51(2.40)	83.91(1.11)	78.79
IRM	83.59(0.15)	77.46(0.39)	73.80(0.09)	90.29(1.63)	72.54(0.29)	68.94(1.65)	75.81(1.93)	83.53(0.91)	66.67(1.47)	84.47(5.49)	83.58(0.74)	78.24
VREX	83.51(0.09)	77.50(0.18)	73,80(0.02)	88.76(2.67)	72.81(0.36)	69.83(1.87)	75.25(2.06)	82.73(1.99)	**67.44(0.58)**	89.56(1.12)	83.38(1.01)	78.6
DIR	83.58(0.19)	**77.74(0.00)**	73.75(0.13)	86.98(6.21)	71.71(0.37)	69.02(0.90)	75.71(3.66)	84.51(2.18)	67.28(0.61)	**91.78(0.00)**	75.41(1.81)	77.95
MoleOOD	83.27(0.13)	77.30(0.58)	73.71(1.31)	87.23(2.80)	71.27(1.66)	66.08(3.78)	**76.38(1.19)**	84.62(1.03)	66.67(0.35)	**91.78(0.00)**	84.80(0.65)	78.46
CIGA	**83.65(0.05)**	77.58(0.23)	73.81(0.04)	89.21(1.38)	72.85(0.95)	69.75(1.02)	**76.38(1.71)**	81.76(2.33)	66.74(1.42)	91.75(0.05)	83.50(1.35)	78.82
iMoLD	**83.65(0.00)**	77.35(1.73)	73.50(0.44)	90.54(0.54)	**73.79(0.05)**	(71.21(0.05))	76.13(1.53)	83.65(1.72)	66.82(0.37)	89.81(1.07)	83.98(0.08)	79.13
CAL-plus	81.73(0.31)	76.93(0.06)	69.64(0.78)	64.11(8.66)	69.50(0.25)	64.11(5.78)	73.90(1.22)	81.82(1.12)	67.06(0.80)	86.6(0.81)	85.21(1.39)	75.6
**CSRL**	83.50(0.00)	77.70(0.00)	73.43(0.15)	**91.07(0.24)**	73.28(0.12)	69.56(1.61)	74.48(1.42)	**84.83(1.00)**	65.89(1.90)	91.00(0.31)	**86.11(0.06)**	**79.17**

© The Author 2022. Published by Oxford University Press. All rights reserved. For permissions, please e-mail: journals.permissions@oup.com

Table S2. The number of positive and negative samples in the test dataset, as well as the accuracy when the model predicts all samples as the majority class (the class with the largest number of samples).

Method	DrugOOD-IC50			DrugOOD-EC50			ADME-OOD-EC50		ADME-OOD-KI		BBBP
	Assay	Scaffold	Size	Assay	Scaffold	Size	Core	Scaffold	Core	Scaffold	
Neg	3176	4336	4388	237	762	765	231	500	291	1105	103
Pos	16287	15144	12373	2488	2000	1740	64	83	140	99	305
ACC	83.68	77.74	73.82	91.3	72.41	69.46	78.31	85.76	67.52	91.78	74.75
